# Analysis of the spatial differentiation and scale effects of the three-dimensional architectural landscape in Xi’an, China

**DOI:** 10.1371/journal.pone.0261846

**Published:** 2021-12-28

**Authors:** Wenjing Ren, Jingyuan Zhao, Xina Ma, Xiao Wang

**Affiliations:** 1 College of Architecture, Chang’ an University, Xi’an, Shaanxi, China; 2 College of Architectural Engineering, Yuncheng Vocational and Technical University, Yuncheng, Shanxi, China; East China Normal University, CHINA

## Abstract

Three-dimensional landscape patterns are an effective means to study the relationship between landscape pattern evolution and eco-environmental effects. This paper selects six districts in Xi’an as the study area to examine the spatial distribution characteristics of the three-dimensional architectural landscape in the city’s main urban area using three-dimensional information on the buildings in 2020 with the support of GIS. In this study, two new architectural landscape indices—landscape height variable coefficient and building rugosity index—were employed in landscape pattern analysis, whilst a system of rigorous and comprehensive three-dimensional architectural landscape metrics was established using principal component analysis. A mathematical model of weighted change of landscape metrics based on the objective weighting method was applied to carry out scale analysis of the landscape patterns. Spatial statistical analysis and spatial autocorrelation analysis were conducted to comprehensively study the differentiation of three-dimensional architectural landscape spatial patterns. The results show that the characteristic scale of the three-dimensional landscape pattern in Xi’an’s main urban area is around 8 km. Moreover, the three-dimensional landscape of the buildings in this area is spatially positively correlated, exhibiting a high degree of spatial autocorrelation whilst only showing small spatial differences. The layout of the architectural landscape pattern is disorderly and chaotic within the second ring, whilst the clustering of patch types occurs near the third ring. Moreover, the building density in the Beilin, Lianhu, and Xincheng districts is large, the building height types are rich, and the roughness of the underlying surface is high, such that these are key areas to be improved through urban renewal. The height, volume, density, morphological heterogeneity, and vertical roughness of the architectural landscape vary amongst functional areas within the study area. This paper is the first to apply the study of spatial heterogeneity of three-dimensional landscape patterns to Xi’an. It does so in order to provide a quantitative basis for urban landscape ecological design for urban renewal and the rational planning of built-up areas, which will promote the sustainable development of the city’s urban environment.

## Introduction

In China’s rapid urbanization process, with economic development and population growth, cities are expanding rapidly, and the artificial landscape continues to expand. As a complex artificial landscape, the vegetation-based natural landscape is gradually replaced by buildings to meet the demand of the expanding population for limited urban land [1, 2]. The current urban spatial form and organizational structure are rapidly developing toward the vertical direction, and the urban architectural landscape is significantly higher than other landscapes, which has a significant impact on the pattern and functional characteristics of the urban landscape. Therefore, the spatial role of structural features and functions of urban landscapes is reflected not only in two-dimensional space but also in the three-dimensional space to show the spatial relationship of urban landscapes. Our discussion on the spatial pattern and functional characteristics of urban landscapes cannot be limited to the two-dimensional plane [3].

Landscape ecology is a rapidly developing discipline of modern ecology and interdisciplinary science [4]. Landscape ecology applies ecosystem principles to study the interactions between landscape patterns and ecological processes, scales, and levels for the ultimate purpose of landscape sustainability [5–7]. Landscape patterns refer to the spatial structure characteristics of the landscape in addition to the type, number, and spatial configuration of landscape component units, which is a comprehensive spatial expression of landscape heterogeneity. The development of methods for quantifying landscape patterns not only helps to understand the interaction of patterns and processes but also has significant implications for applying the concepts of landscape ecology to sustainable landscape planning [8, 9]. Landscape pattern metrics are commonly used quantitative methods for landscape pattern analysis [10]. They can condense landscape pattern information, reflect its structural composition and spatial configuration characteristics and are a powerful tool for scientifically describing the relationship between landscape patterns and ecological processes [11]. Landscape metrics are widely used in spatial pattern analysis, making the landscape planning process more accurate, objective, and reliable [12]. This study analyzed the 3-D landscape pattern of the main urban area of Xi’an by constructing a 3-D urban landscape metrics system.

The acquisition of urban 3-D information is the premise and foundation of urban 3D landscape research. Aerial photogrammetry, high-resolution remote sensing satellite images, airborne laser scanning and 3-D cadastral data provided the main data sources for obtaining 3-D urban landscape information, among which aerial photogrammetry and satellite telemetry mainly acquired the top surface of buildings and their contours [13–15]. Airborne laser scanning technology can obtain both ground and surface elevation information through multiple echo detection, yet the performance of 2-D features is poor [16]. 3D cadastral data are time-consuming and laborious to acquire [17]. Therefore, this study made use of the 3-D data of buildings from the China Geography Census database and constructed a multisource dataset to analyze the 3-D landscape pattern of the main urban area of Xi’an.

As an important carrier of urban physical space, architecture is the core element of urban landscapes. Quantifying three-dimensional landscape patterns is an effective way to study the relationship between the evolution of landscape patterns and coenvironmental effects [18]. To characterize urban 3-D landscape change more comprehensively, researchers have used a variety of landscape indices to study the spatial and temporal patterns of urban landscapes [19]. In recent years, many studies have tried to reveal the spatial characteristics of urban landscapes based on remote sensing data [20, 21]. The study found that the study of 2-D urban landscapes can no longer meet the needs of urban vertical expansion, and revealing the differentiation of 3-D urban landscape patterns is more conducive to the study of urban landscapes and their ecological effects [22]. Based on the construction of the landscape metrics system, some researchers have studied the spatial heterogeneity analysis of the 3-D urban landscape pattern and found that the metrics constructed with 3-D architectural data can well reflect the changing characteristics of the architectural landscape [23]. However, these studies rarely analyze the validity of index selection. Researchers have also paid much attention to the scale effect of urban landscape patterns and found that there is a grain size effect and extent effect of urban landscape patterns [24, 25]. However, there are fewer research results on the magnitude effect, especially in Xi’an, where no research has been conducted. Therefore, compared with previous studies, this study used principal component analysis to screen the indices and enhance the validity of metric selection. By introducing two new landscape indices, new possibilities for the selection of landscape metrics were provided. In addition, the extent effect of the landscape pattern in the main urban area of Xi’an was analyzed by establishing a mathematical model of the weighted change in the landscape metrics based on the objective assignment method. This study fills the gap in the research on the magnitude effect of the 3-D landscape in Xi’an.

The purpose of this study was to construct a metric system for the 3-D architectural landscape pattern in the study area, establish a mathematical model for the weighted change in landscape metrics based on the objective weighting method, and reveal the characteristic scale of the 3-D architectural landscape pattern in the main urban area of Xi’an. In addition, it aims to provide a quantitative basis for urban landscape ecological design for urban renewal and rational planning of built-up areas by studying the differentiation characteristics of the 3-D architectural landscape pattern. Finally, it provides important support for further research on the mechanism by which 3-D landscape patterns influence the urban ecological environment, which has a positive role in promoting the sustainable development of the urban environment.

## Materials and methods

### Study area

**Fig 1:**
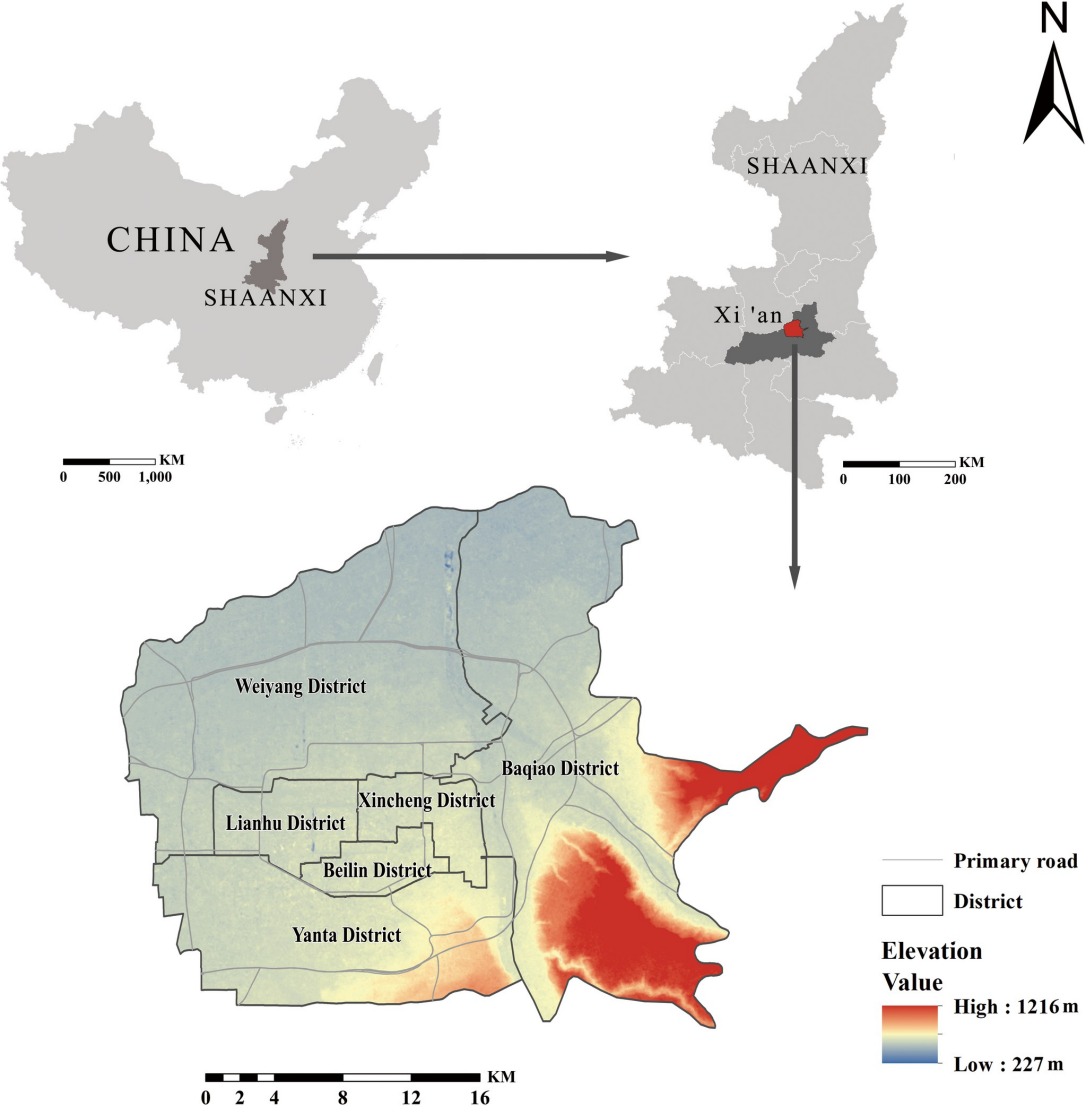
Location of study area. The map images are from the National Catalogue service for Geographic Information: http://www.ngcc.cn/.

Xi’an, the capital of Shaanxi Province and an important central city in western China, is located in the Guanzhong Basin in the middle of the Yellow River watershed in eastern Northwest China, between 107°40’-109°49’ east longitude and 33°42’-34°45’ north latitude; it is adjacent to the Weihe River and Loess Plateau in the north and the Qinling Mountains in the south [26]. The jurisdiction is approximately 204 km long from east to the west and 116 km wide from north to south. It has an area of 10,096 km^2^, of which 5,145 km^2^ represent the urban area. Xi’an is composed of 11 districts with two counties. The study area is the main urban area of Xi’an, namely, Beilin District, Yanta District, Lianhu District, Xincheng District, Baqiao District and Weiyang District, as shown in Fig 1. According to the Xi’an Statistical Yearbook, the population density of these six districts is greater than 15,000 people/km^2^. With accelerated urbanization, the population of Xi’an is increasing, especially in the main urban area, and building density are constantly increasing, which brings severe challenges to the resources and ecological environment of the area [27].

### Data

#### Administrative divisions and road network information

The administrative zoning data were mainly collected from the National Geomatics Center of China, including data related to national boundaries, provincial boundaries, municipal boundaries and district boundaries. The road network data were extracted from OpenStreetMap for five types of road networks: motorways, primary roads, secondary roads, tertiary roads, and trunk lines.

#### Land use information

**Fig 2:**
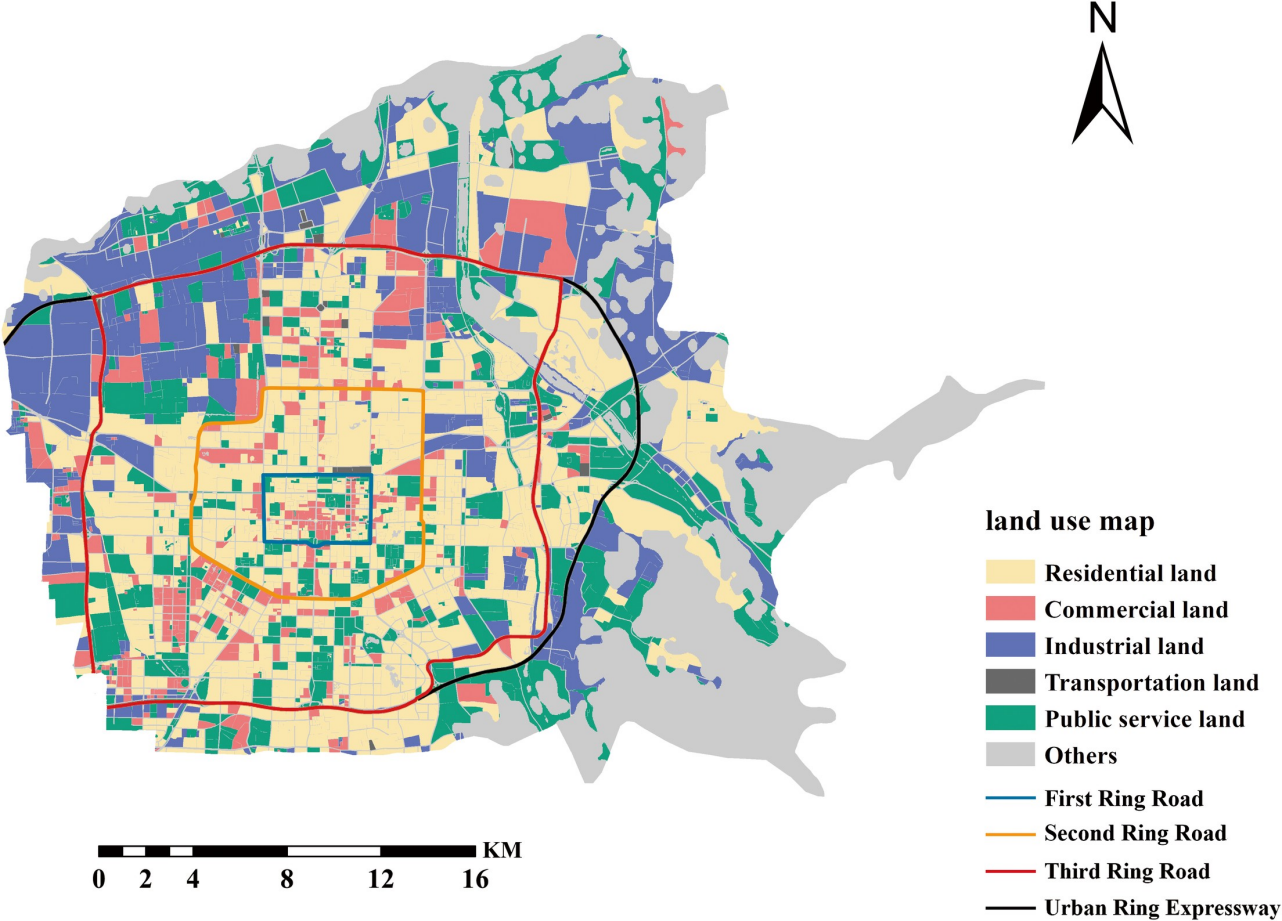
Land use map of the study area. The map images are from the National Catalogue service for Geographic Information: http://www.ngcc.cn/.

Landsat TM/ETM/OLI remote sensing images were the main data source. After preprocessing, such as orthorectification and image fusion, remote sensing images with a 30 m spatial resolution of Xi’an city were obtained. Then, object-oriented classification and visual interpretation methods were used to extract remote sensing image information and obtain land cover classification data. Combining land use coverage types and urban functions, the land use of the study area was classified into five categories: residential land, commercial land, industrial land, transportation land, and public service land, as shown in Fig 2.

#### 3-D Architectural information

The 3-D architectural data used in this study were obtained from the National Geomatics Center of China’s Geographic National Conditions database (http://www.ngcc.cn/). The database includes the data of national geographic census results and the data of basic monitoring results carried out in previous years. The building data in the database were primarily collected using high-resolution satellite images, combined with geographic mapping, automatic computer classification, and manual interpretation. The 3-D building data of Xi’an in the 2019 Geographical National Conditions database obtained by application to the natural resources management department include height information. According to the high-resolution remote sensing images in 2020, the visual interpretation of buildings was carried out on the basis of the acquired data, and the number of buildings was added or subtracted. Then, the mathematical relationship between building shadows and actual building heights in the high-resolution remote sensing images was used to obtain the building distribution data in 2020. The total accuracy rate reached 88.13% and was verified by field measurement data, which was suitable for 3-D architectural landscape recognition. In this study, buildings were classified into five types according to their heights, low-rise buildings (≤9 m), multistory buildings (10 < H ≤ 18 m), mid-rise buildings (18 < H ≤ 30 m), high-rise buildings (30 < H ≤ 100 m) and super high-rise buildings (>100 m), and these five building types participated in the calculation of the architectural landscape metrics as patch types.

### Metrics

#### Index selection

Five categories of urban architectural landscape metrics were introduced, including a total of 12 landscape level indices. Among them were height metrics, including average height (AH), landscape height standard deviation (LHSD), and landscape height variation coefficient (LHVC). Quantity/density metrics, including number of buildings (N), high building ratio (HBR), building coverage ratio (BCR), and floor area ratio (FAR). Shape metrics were average volume (AV), building shape coefficient (BSC) and building rugosity index (BRI). Contagion index (CONTAG) was selected for aggregation/dispersion metrics, and Shannon diversity index (SHDI) was selected for diversity metrics. In this study, to analyze the 3-D spatial structure and heterogeneity of the architectural landscape more comprehensively, the landscape height variation coefficient and the building rugosity index were created for the first time.

The architectural landscape metrics were replaced by abbreviations in the latter part of the text. The architectural landscape metrics were calculated in ArcGIS 10.6, except for CONTAG and SHDI, which were calculated with the landscape metrics calculation software Fragstats 4.2 [28].

#### Principal component analysis (PCA)

A geographic information system was a complex system with multiple variables. Too many variables undoubtedly increase the difficulty of problem analysis. Therefore, if the original variables can be replaced by fewer independent variables and as much information of the original variables as possible can be retained, then the problem will be simplified. Principal component analysis was a dimensionality reduction process to solve such problem. PCA was adopted in this research to exclude the overlapping parts of the numerous landscape information so that the selected variables could represent the landscape information contained in the original multiple variables as much as possible. In this PCA, a sample block of 2.8 km × 2.8 km from the land cover data of Xi’an in 2020 was adopted, and a total of 81 samples were used to analyze 12 variables.

**Fig 3:**
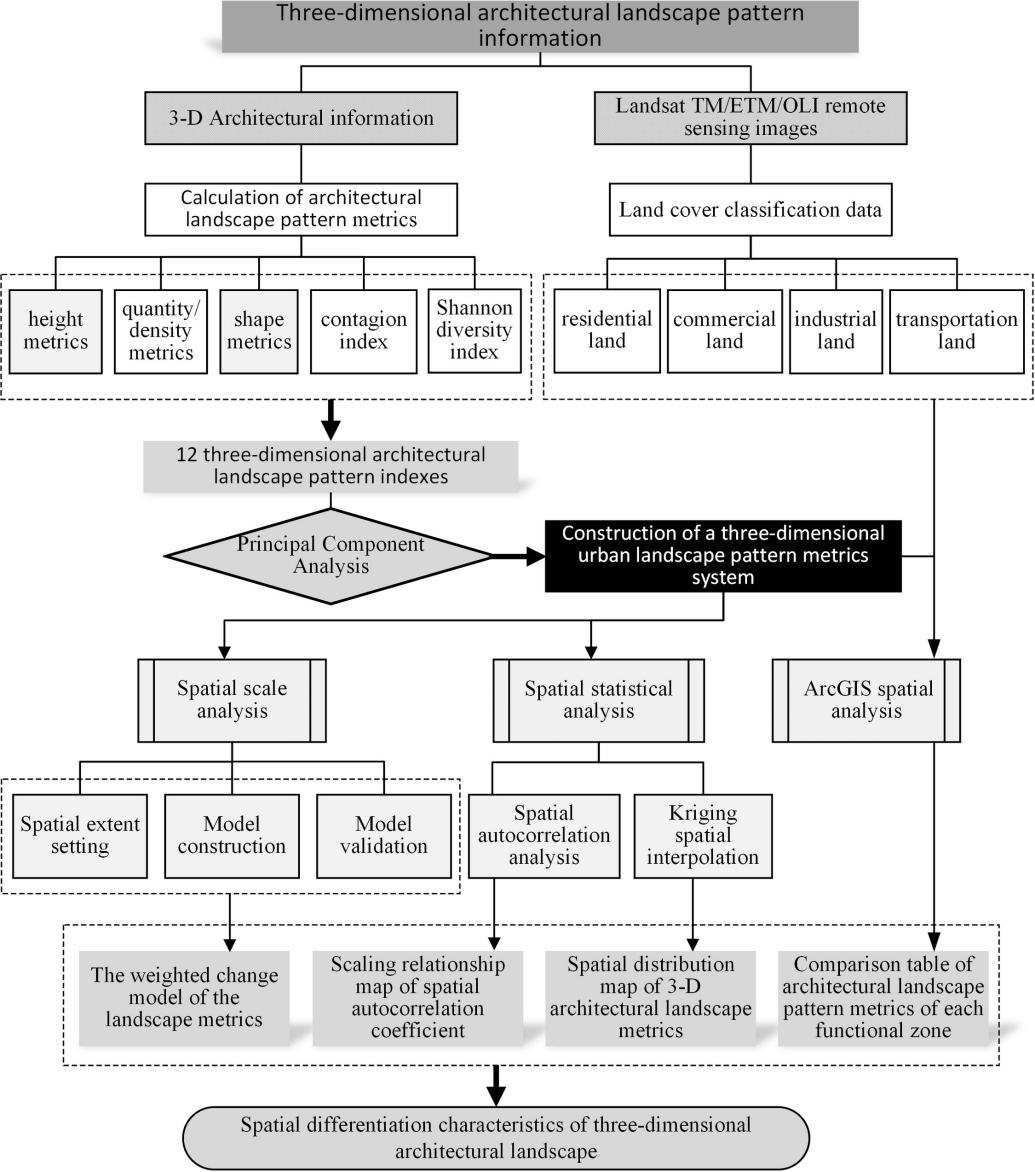
Research flow chart.

The framework of establishing and analyzing of the architectural 3-D landscape metrics system is shown in Fig 3.

### Spatial scale analysis

#### Spatial extent setting

**Fig 4:**
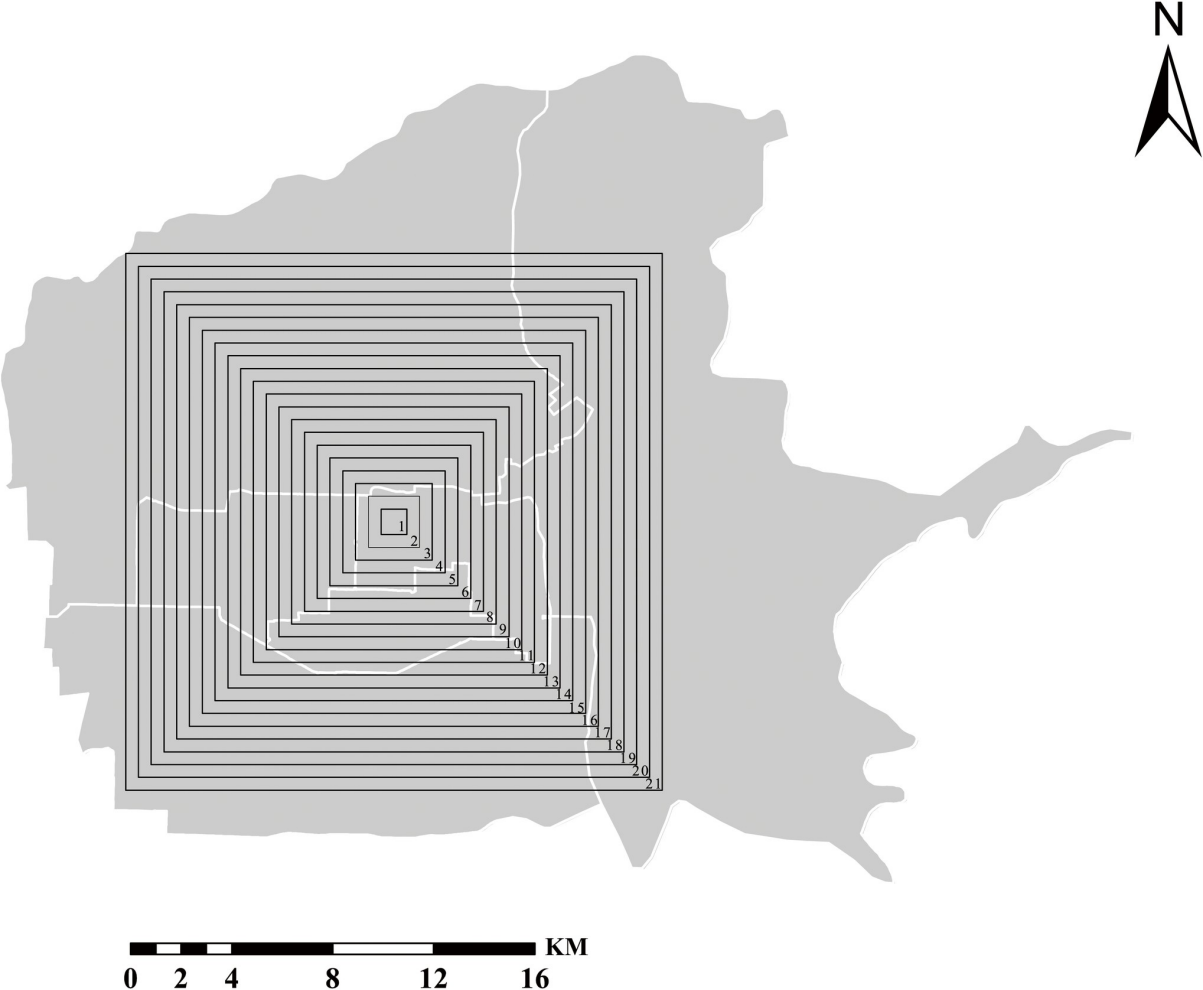
Setting of extent variation. The map images are from the National Catalogue service for Geographic Information: http://www.ngcc.cn/.

The spatial scale was generally divided into grain size and extent. For spatial data or image data, the grain size corresponds to the maximum resolution or pixel size. The extent was determined by the size of the study area. If the scale is not chosen properly, the nature of the object of study cannot be scientifically revealed. If the scale chosen is too large, a large number of details are often ignored, making the Study a "biased" estimation. If the scale is too small, it is easy to ignore the overall pattern. If the scale conversion restriction was ignored and the scale conversion was done arbitrarily, the conclusions obtained were often biased. In this study, the grain size at a 30 m spatial resolution was kept unchanged, and the effect of scale change on the landscape metrics was studied through extent change. The spatial extent of the scale analysis was set by a square of 1 km×1 km in the geometric center of the study area with a step length of 500 m gradually increasing outward. The minimum extent was 1 km×1 km, and the maximum extent was 21 km×21 km, as shown in Fig 4.

#### Model construction

To study the characteristic scales of the landscape pattern in the main urban area of Xi’an, this study comprehensively analyzed the scale effect of the landscape pattern with extent change by calculating the weight of each landscape index in the 3-D landscape pattern.

The subjective weighting method relies too much on expert experience and deviates from actual data characteristics. In contrast, the entropy method commonly used in the objective weighting method can calculate the weight by using the amount of information carried by data [29]. However, in the entropy method, the correlation information between different data series and the dynamic changes of the overall data cannot be well represented [30]. To overcome the above problems, in this study, we used the CRITIC method to calculate the weights of landscape indices. CRITIC (criteria importance through intercriteria correlation) is an objective weighting method based on the evaluation of the contrast strength of indices and the conflict between indices to measure the objective weight of indices. It takes into account the correlation between indices while considering the magnitude of index variability and uses the objective properties of the data itself for scientific evaluation [31]. In the CRITIC method, the standard deviation was used to indicate the fluctuation of the difference between the values of each index. The larger the standard deviation is, the greater the difference is in the values of the index exist, the more information can be reflected, and the stronger the evaluation intensity of the index itself; then, more weight should be assigned to the index. At a certain standard deviation, the smaller the conflict between indices is, the smaller the weight is. The larger the conflict is, the larger the weight is. The basic steps are as follows.

Assuming that there are *n* samples to be evaluated and *p* evaluation indices, the original index data matrix is formed:

$$X=(\begin{array}{ccc} x_{11} & \ldots & x_{1p}\\ \vdots & \ddots & \vdots \\ x_{n1} & \cdots & x_{np} \end{array})$$

where *x*_*ij*_ represents the value of the *j*-th evaluation index for the *i*-th sample.

To eliminate the influence of different dimensions on the evaluation results, it was necessary to conduct dimensionless processing for each index, as shown in the following equation.


$$x_{ij}^{\prime }=\frac{x_{j}-x_{\min}}{x_{\max}-x_{\min}}$$ (1)

The variability of the index is expressed in the form of standard deviation:

$$\{\begin{array}{l} {\bar{x}}_{j}=\frac{1}{n}\sum_{i=1}^{n}x_{ij}^{\prime }\\ S_{j}=\sqrt{\frac{\sum_{i=1}^{n}{\left(x_{ij}^{\prime }-{\bar{x}}_{j}\right)}^{2}}{n-1}} \end{array}$$ (2)

*S*_*j*_ represents the standard deviation of the *j*-th index.

The conflict of the indices is expressed in terms of correlation coefficients:

$$R_{j}=\sum_{i=1}^{p}\left(1-r_{ij}\right)$$ (3)

*R*_*j*_ represents the conflict of the *j*-th index; *r*_*ij*_ represents the correlation coefficient between the evaluation index *i* and *j*.

The amount of information is as follows:

$$C_{j}=S_{j}\sum_{i=1}^{p}\left(1-r_{ij}\right)=S_{j}\times R_{j}$$ (4)

*C*_*j*_ represents the information content of the *j*-th index, the larger *C*_*j*_ is, the greater the role of the *j*-th evaluation index in the whole evaluation metrics system, and the more weight should be assigned to it.

The objective weights *W*_*j*_ of the *j*-th index are the following equation:

$$W_{j}=\frac{C_{j}}{\sum_{j=1}^{p}C_{j}}$$ (5)

In this study, the above calculation process of the CRITIC method was completed in MATLAB software.

On this basis, this study obtained the weighted average of the total change in the landscape metrics at different scales by superimposing the extent of the change in the standardized landscape index at each spatial scale interval. The weighted change mathematical model of the landscape metrics is shown in the following equation.

$${\bar{y}}_{i}=\frac{\sum_{j=1}^{n}\left(|x_{ij}-x_{(i-1)j}\right|\times W_{j})}{\sum_{j=1}^{n}W_{j}}$$ (6)

${\bar{y}}_{i}$ is the weighted average of the total variation of the landscape metrics at scale *i*; *i* ∈2, 3,…, *m*; *j*∈1, 2, …, *n*; *m* is the number of spatial extents; *n* is the number of landscape indices, and *x*_*ij*_ is the normalized value of landscape index *j* at scale *i*.

In this study, the changes in several landscape indices with spatial scales were aggregated into a single index of total variation extent. The scale effect of the landscape metrics was explored according to the total variation extent, and the scale relationship map was used to detect the hierarchical structure and scale characteristics of landscape patterns in the main urban area of Xi’an.

#### Model validation

Fractal analysis is an important method of spatial scale analysis. By analyzing the variation of fractal dimension at different scales, the self-similarity, scale invariance or scale dependence can be detected, and the characteristics of hierarchical structure or fractal structure of landscape pattern process variables, thereby playing a similar role to spatial statistics method.

The fractal dimension calculated using the double logarithmic regression fractal dimension method of perimeter-area is equivalent to FAFRAC in Fragstats software. This regression method considers patches of different sizes, and the resulting fractal dimension can reflect the pattern characteristics at different scales.

$$\mathrm{PAFRAC}=\frac{\frac{2}{\left[n_{i}\sum_{j=1}^{n}\left(\ln p_{ij}\cdot \ln a_{ij}\right))-[\left(\sum_{j=1}^{n}\ln p_{ij})(\sum_{j=1}^{n}\ln a_{ij}\right)\right]}}{(n_{i}\sum_{j=1}^{n}\ln p_{ij}^{2})-{\left(\sum_{j=1}^{n}\ln p_{ij}\right)}^{2}}$$ (7)

*P*_*ij*_ is the perimeter of patch *ij*, *a*_*i*j_ is the area of patch *ij*, and *n*_*i*_ is the number of patches of patch type *i*. *P*_*ij*_ reflects the shape complexity of a series of patches of different sizes; the larger the value is, the more complex the shape is.

In this paper, Fragstats software was used to calculate the fractal dimension of different scales to verify the weighted change model of landscape metrics.

### Spatial statistical analysis

#### Spatial autocorrelation analysis

In the process of development and urban reconstruction of Xi’an, the landscape pattern changed, and spatial heterogeneity was obvious. In this study, the Moran index was mainly applied to reflect the degree of spatial autocorrelation in the spatial analysis. The Moran index reflects the similarity degree of attribute values of spatially adjacent regional units. The global Moran index *I* was calculated by the following equation.

$$I=\frac{n\sum_{i=1}^{n}\sum_{j=1}^{n}\omega_{ij}\left(x_{i}-\bar{x}\right)\left(x_{j}-\bar{x}\right)}{\sum_{i=1}^{n}\sum_{j=1}^{n}\omega_{ij}\sum_{i=1}^{n}{\left(x_{i}-\bar{x}\right)}^{2}}$$ (8)

where *x*_*i*_ and *x*_*j*_ respectively represent the values of landscape elements in adjacent pairs of spatial units, $\bar{x}$ are the mean values of the variables, *n* is the total number of spatial units, and *ω*_*ij*_ is the neighboring weight. When spatial units *i* and *j* are adjacent, *ω*_*ij*_ = 1, and when spatial units *i* and *j* are not adjacent, *ω*_*ij*_ = 0.

The Moran index *I* had a value range of [–1, 1], and the larger the absolute value of the Moran index is, the greater the correlation is. When *I* > 0 was spatially positively correlated, when *I* < 0 was negatively correlated, and when *I* = 0 was significantly spatial uncorrelated.

In this paper, based on the weights of each index obtained by the CRITIC weighting method, a new variable representing the comprehensive information of the architectural landscape pattern was obtained. The Moran index of the new variables was calculated to analyze the degree of spatial autocorrelation of the architectural landscape pattern in the study area to map the scale relationship of the spatial autocorrelation coefficients.

#### Kriging spatial interpolation

The spatial heterogeneity analysis of 3-D architectural landscape patterns was mainly realized by the kriging spatial interpolation method. Kriging’s interpolation method is based on the condition that the regionalized variables are spatially correlated. In essence, it uses the measured data of the regionalized variables and the structural characteristics of the variation function to make linear unbiased and optimal estimates of the values of the regionalized variables at the unsampled points. The variogram model used in this interpolation is the spherical model, which is the most widely used theoretical model. In this study, ArcGIS was used to divide the study area into 3480 cells, each with an area of 500 m×500 m. All landscape metrics were calculated and connected with all the cells. Finally, kriging spatial interpolation was carried out in ArcGIS.

In addition to the above methods, finally, the land use of the study area was functionally zoned according to the land use data of Xi’an in 2020. The regional statistics function of ArcGIS spatial analysis was used to count the landscape pattern indices in different functional areas and make regional comparisons.

## Results

### 3-D architectural landscape pattern metrics system

SIMCA software was researched and developed by the Swedish company Umetrics in 1987. Multivariate statistical analysis software excels in performing principal component analysis and partial least squares discriminant analysis on datasets with many variables and interpreting the data results. In this paper, 12 variables with 81 samples were initially selected, the data were standardized and analyzed by PCA with SIMCAI 14.1, and the results are shown in Table 1.

**Table 1:** Analysis results with SIMCA software.

Component	R2X	R2X(cum)	Eigenvalue	Q2	Limit	Q2(cum)	Significance
1	0.407	0.407	4.88	0.245	0.0883	0.245	R1
2	0.252	0.659	3.02	0.29	0.0948	0.465	R1
3	0.124	0.783	1.49	-0.044	0.102	0.441	R2

The result of the PCA model of the 3-D architectural landscape shows that the number of principal components was 3, which means that the analysis shows that there are three principal components that were the most effective. R2X represents the explanation rate corresponding to each component. R2X(cum) represents the cumulative explanation rate at the current number of principal components, and the results show that the cumulative explanation rate of three principal components for 12 variables of the 3-D architectural landscape reaches 78.3%, i.e., three principal components can explain the variables well. Q2 represents the predictive power corresponding to each component, while Q2(cum) represents the cumulative value of the predictive power of the principal components. The eigenvalue represents the load value corresponding to each component, and the load value represents how many variables each principal component holds. The eigenvalue of the three principal components was 9.39, which means that they can explain 9.39 out of 12 variables. The value of limit corresponds to the critical value when Q2 of the principal component was not significant, and the model result was significant if it was higher than this value. Significance refers to whether cross-validation was significant, and the result for R1 show there is very significance. The results for R2 indicate significance. The results of Q2 show that the first and second principal components were highly predictive, while the third principal component was poorly predictive.

**Fig 5:**
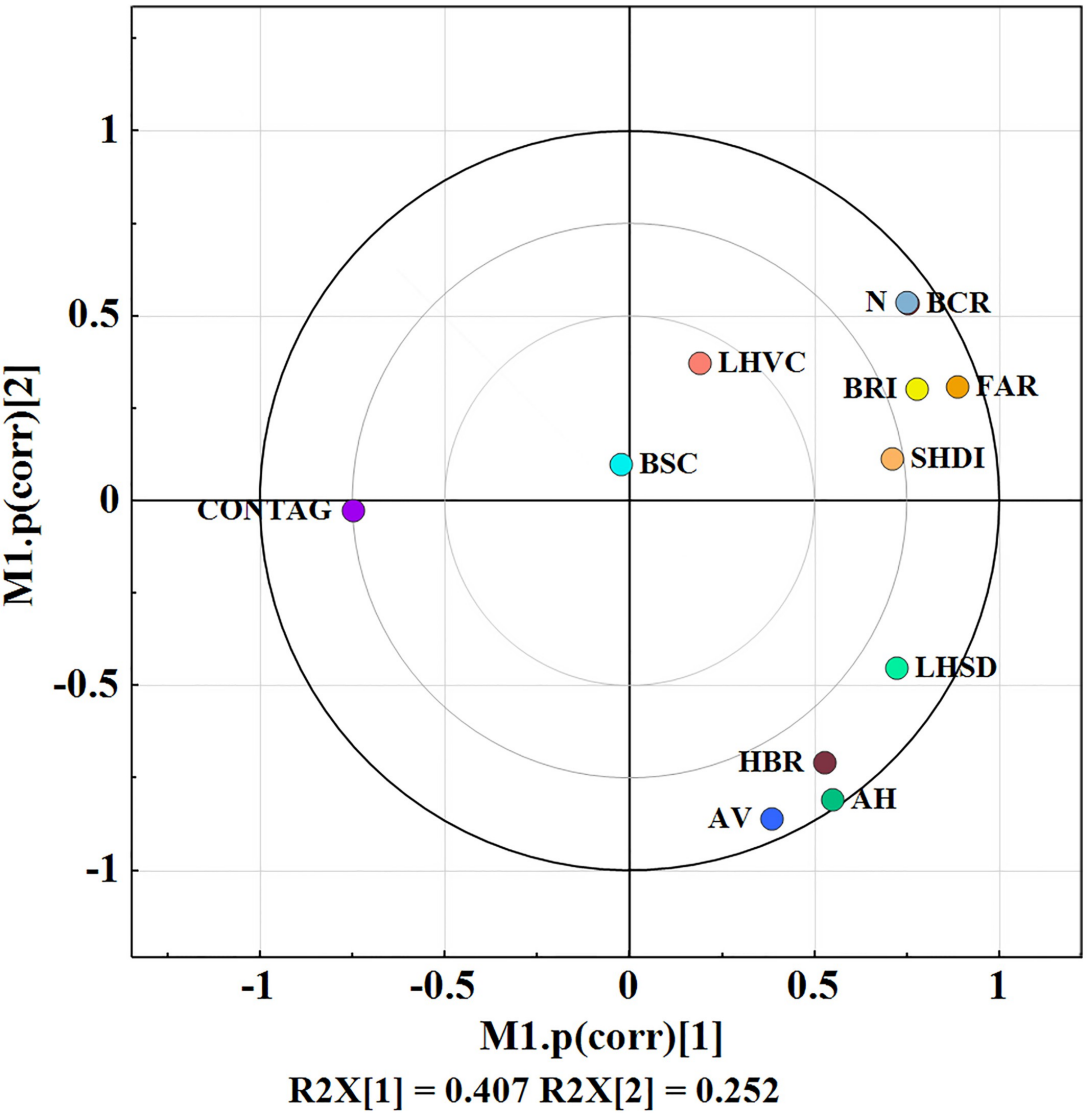
Load plot of variables.

The load plot in SIMCA can also be called the correlation plot, where variables with strong correlations were aggregated together, variables with opposite correlations were distributed at both ends of the line passing through the origin, and variables were independent of each other at an angle of 90 degrees. The "magnitude or quality" of the variable’s influence on the sample can be evaluated on the load plot by finding the distance between the variables and the origin. Fig 5 shows that there is an obvious aggregation of variables in the upper right and lower right.

The purpose of this PCA is to select independent indices and eliminate those with large redundancy. Therefore, on the premise of maintaining independence among variables, the contribution degree of variables to principal components was considered. The building height metrics contain AH, LHSD and LHVC. From the load diagram, it could be seen that the AH load value was larger and positively correlated with the LHSD, but they were weakly correlated with the LHVC. Therefore, AH with large load values and LHVC were selected. The quantity/density metrics contain four indices, N, BCR, HBR and FAR. N, BCR and FAR were positively correlated, and the two indices of N and BCR were basically independent of HBR. The two indices of BCR and HBR with larger loads were finally selected. The shape metrics contain three indices, AV, BSC and BRI. A low load value of BSC was positively correlated with BRI and negatively correlated with AV. BRI and AV were basically independent of each other, and these two indices were retained. CONTAG and SHDI represent the connectivity and diversity of the landscape, respectively, and CONTAG shows great differences from other indices; thus, both of these indices were retained. Finally, the architectural landscape pattern retained eight indices: AH, LHVC, BCR, HBR, AV, BRI, CONTAG and SHDI.

Detailed explanations and sources of CONTAG and SHDI can be found in the Fragstats user help manual. The specific formulas and parameter meanings of the other indices are shown in Table 2. Therefore, a 3-D architectural landscape metrics system composed of eight indices was constructed to analyze the spatial pattern of the architectural landscape in the main urban area of Xi’an.

**Table 2:** Index of terms and their meanings.

Metrics type	Index name	Formula	Description
Height metrics	Average heigh (AH)	$AH=\frac{1}{n}\sum_{i=1}^{n}H_{i}$	This reflects the overall level of urban building heights versus the expansion of the city in the vertical direction.
	Landscape height variable coefficient (LHVC)	$LHVC=\frac{1}{AH}\sqrt{\frac{1}{n-1}\sum_{i=1}^{n}{\left(H_{i}-AH\right)}^{2}}$	This reflects the relative change degree of the height of urban architectural landscape within a certain range.
Quantity/density metrics	Building coverage ratio (BCR)	$BCR=\frac{1}{A}\sum_{i=1}^{n}F_{i}$	This reflects the open space ratio and building density of a certain land area.
	High building ratio (HBR)	*HBR* = *N*_*h*_/*n*	This index represents the proportion of buildings over 24 m in height.
Shape metrics	Average volume (AV)	$AV=\frac{1}{n}\sum_{i=1}^{n}V_{i}$	This reflects the size of the building volume and space heat loss area and the amount of energy consumption.
	Building rugosity index (BRI)	$BRI=\sum_{i=1}^{n}(1+\frac{P_{i}\times H_{i}}{F_{i}})$	This reflects the roughness of the ground surface within a certain range.
aggregation/dispersion metrics	Contagion index (CONTAG)	$CONTAG=[1+\frac{\sum_{i=1}^{m}\sum_{k=1}^{m}\left[P_{i}^{{}^{\circ}}\frac{g_{ik}}{\sum_{k=1}^{m}g_{ik}}\right]\cdot [\ln(P_{i}^{{}^{\circ}}\frac{g_{ik}}{\sum_{k=1}^{m}g_{ik}})]}{2\ln(m)}](100)$	*g*_*ik*_ is the number of patches of type *i* and *k* adjacent to each other. *m* is the number of patch types. This reflects the degree of clustering or extension of buildings in the landscape.
diversity metrics	Shannon diversity index (SHDI)	$SHDI=-\sum_{i=1}^{n}P_{i}\times \ln P_{i}$	This reflects the landscape heterogeneity and is sensitive to the uneven distribution of patch types in the landscape.

Note: *Hi*, *Vi*, *Fi* and *Pi* in the formula of *AH*, *LHVC*, *BCR*, *HBR*, *AV* and *BRI* are the height, volume, floor area and underside perimeter of the *i*-th building in a district, respectively; *n*, *AH* is the numbers and average height of buildings, respectively; *A* is the total area of the district*; N*_*h*_ is the number of buildings with a height of more than 24 m. *Pi* in the *CONTAG* and *SHDI* are the percentage of area occupied by type *i* patches.

### Scale effect of 3-D architectural landscape

#### Scale effect of metrics

**Fig 6:**
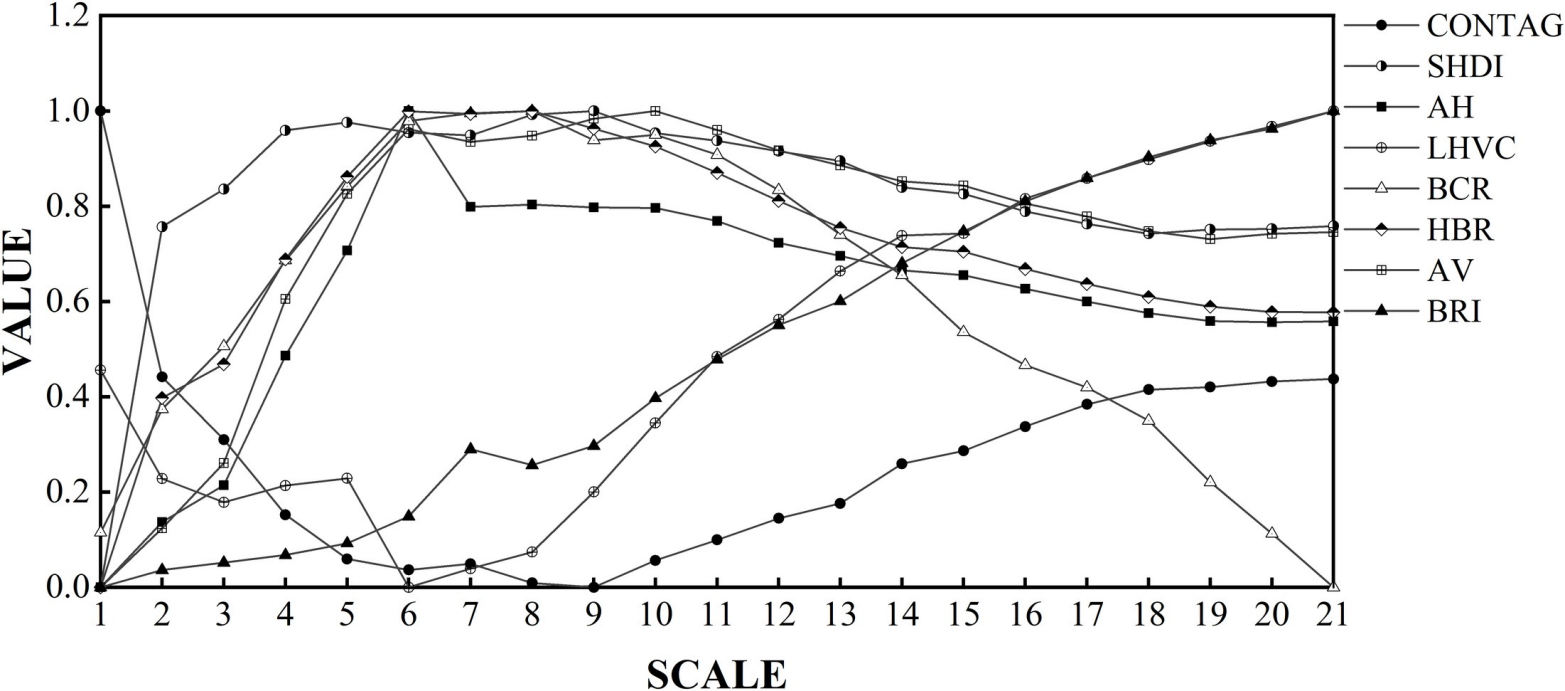
Scale-effect plot of eight architectural landscape pattern indices.

Based on the 500 m×500 m grid, 3-D architectural landscape pattern metrics were calculated to 21 kinds of extent with a grain size of 30 m spatial resolution. After data standardization processing, the response of architectural landscape pattern metrics to extent change was obtained, as shown in Fig 6. The results of the scale effects of the architectural landscape metrics show that the LHVC, BRI and CONTAG have similar trends with the extent and show obviously opposite trends with the other five indices. The overall change in the eight landscape indices shows the following characteristics: a significant change occurs near 6 km and a stable change trend occurs after 8 km.

**Fig 7:**
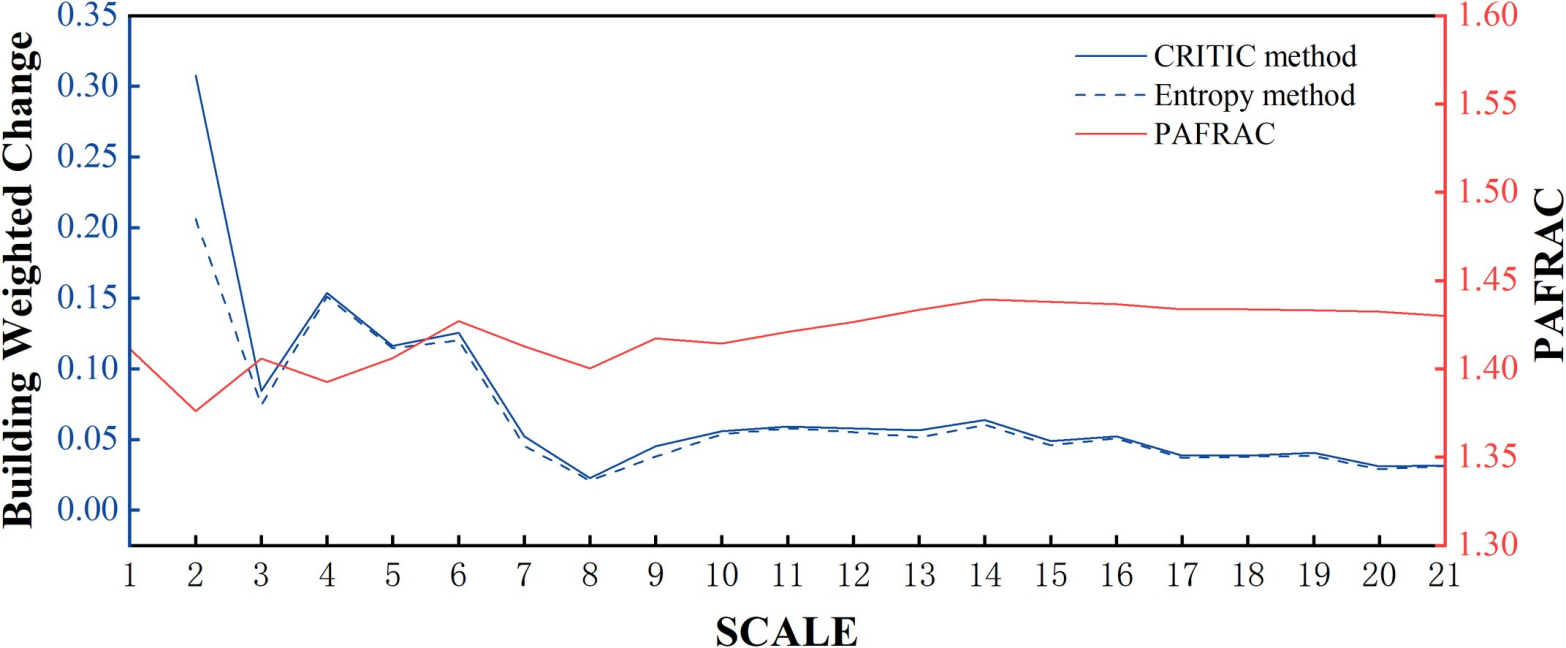
Scale-effect plots of weighted changes in landscape metrics and fractal dimension.

This study used the CRITIC method to calculate the weights of eight indices of architectural landscape patterns. In addition, the weights of the architectural landscape index were calculated using the entropy method, and the results were compared with those of the CRITIC method to observe the robustness of the CRITIC method. The results calculated by MATLAB software are shown in Table 3. Based on the results of the CRITIC method and entropy method, the weighted average of the total change in the extent of the architecture landscape pattern metrics was calculated by Eq (6), and the relationship between this value and the change in extent is shown in Fig 7. The results show that the total change trend of the architecture landscape pattern clearly turns at 3 km, 4 km, 6 km and 8 km and stabilizes after 8 km. The results included the scale effect of the architecture landscape metrics but reflected more abundant change information.

**Table 3:** Weighting values of the eight architectural landscape pattern indices.

	SHDI	CONTAG	AH	BCR	AV	BRI	LHVC	HBR
CRITIC method	0.1175	0.1105	0.0875	0.1749	0.09	0.1482	0.1439	0.1274
Entropy method	0.0287	0.0861	0.1290	0.1440	0.1222	0.3370	0.0396	0.1133

#### Model validation

To verify the mathematical model of the weighted change in landscape metrics based on the CRITIC method, the fractal dimension of the 3-D architectural landscape pattern was calculated using Fragstats software, and the relationship between it and the change in extent is shown in Fig 7. The results show that the fractal dimension PAFRAC of the architecture landscape pattern changes significantly within 8 km, such as a great turning point at 2 km, 3 km, 4 km, 6 km, 8 km, etc., and it gradually becomes stable after 8 km.

The result was basically consistent with the scale effect of the weighted change of the building landscape metrics, which embodies richer information. In summary, it can be concluded that the mathematical model of the weighted change in landscape metrics based on the CRITIC method are effective.

#### Spatial correlation analysis

**Fig 8:**
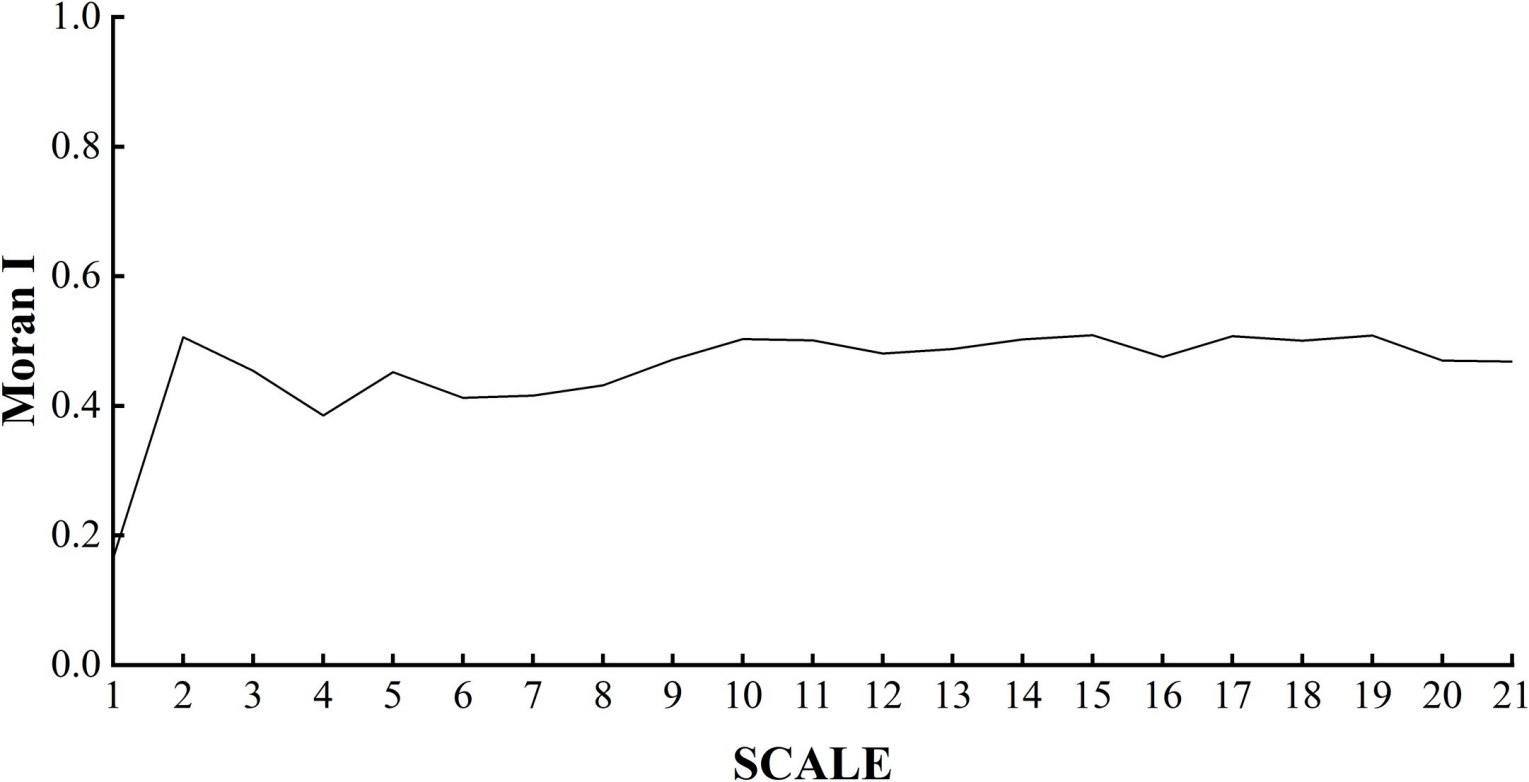
Scale relationship plot of the Moran index of architectural landscape pattern.

The Moran index was calculated for the weighted architectural landscape pattern using the statistical analysis module in ArcGIS, and the relationship between the Moran index and the change in extent was obtained as shown in Fig 8.

The larger the Moran index is, the smaller the spatial variation is; the smaller the Moran index is, the larger the spatial variation is. Near the turning point from positive to negative, the landscape does not have spatial autocorrelation but has a random distribution characteristic. The trend of the Moran index of the architectural landscape pattern with extent shows that the architectural landscape was spatially positively correlated to all extents, with a high spatial autocorrelation and small spatial differences.

### Spatial distribution characteristics of the 3-D architectural landscape

**Fig 9:**
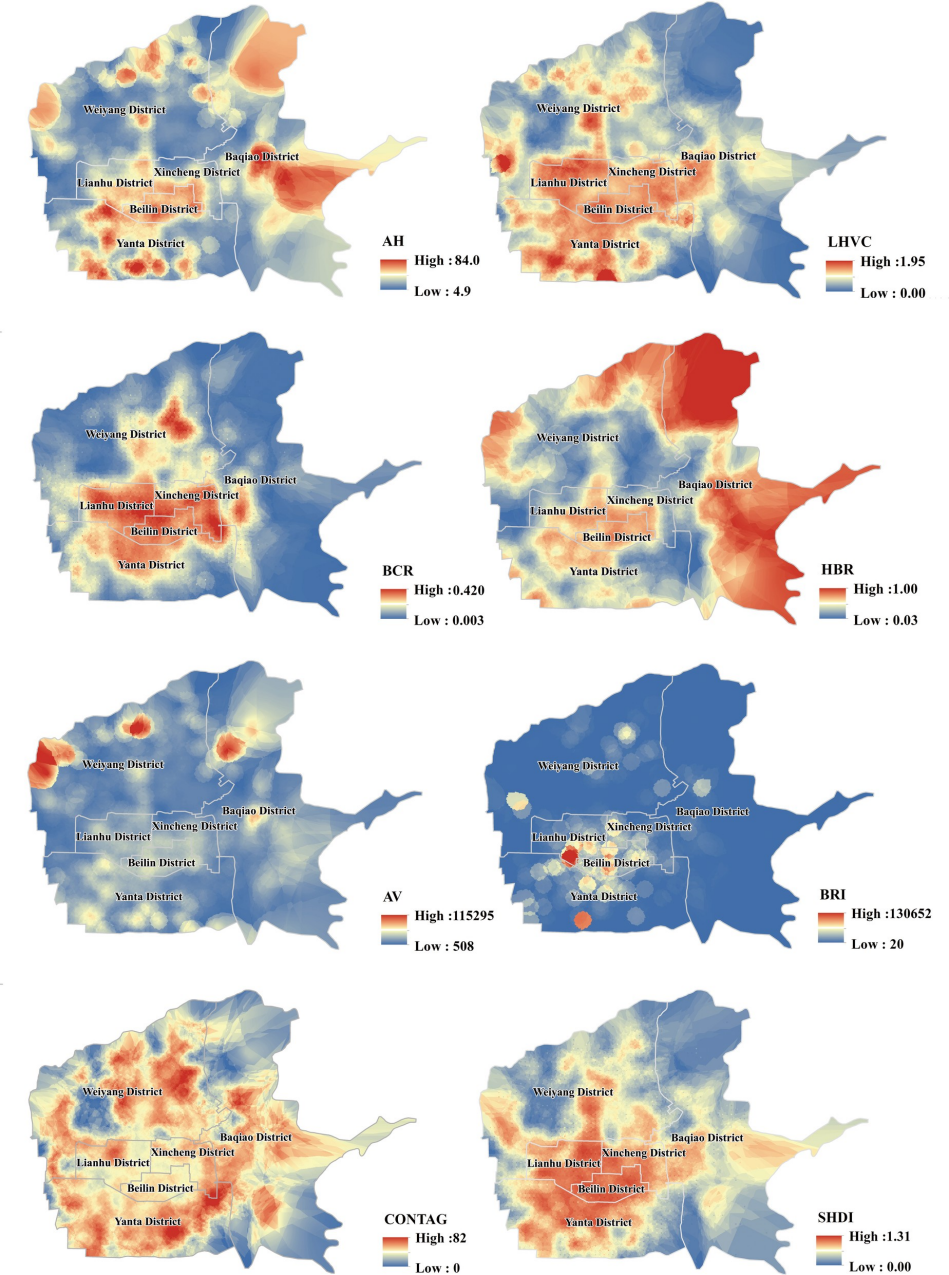
Spatial distribution map of architectural landscape pattern metrics. The map images are from the National Catalogue service for Geographic Information: http://www.ngcc.cn/.

In this study, spatial distribution maps of architectural landscape metrics were generated using ArcGIS visualization, and the results show that there was significant spatial heterogeneity in the 3-D landscape pattern of the main urban area of Xi’an, as depicted in Fig 9. The architectural landscape metrics were calculated for each functional land in the study area using Fragstats software, and the calculated results are presented in Table 4. The results show that there are obvious differences in the architectural landscape patterns of different functional lands.

**Table 4:** Architectural landscape index values for each functional land.

Region	AH	LHVC	BCR	HBR	AV	BRI	CONTAG	SHDI
Residential land	34	1.3112	0.3487	0.3744	22349	9019667	39.2159	1.4052
Commercial land	34	1.3154	0.4600	0.3995	26905	2335524	40.4754	1.4092
Industrial land	19	1.4970	0.1649	0.1458	14627	850781	55.7298	1.1284
Transportation land	32	1.1538	0.3368	0.4107	23961	320348	39.6788	1.4076
Public service land	28	1.2616	0.1996	0.3599	20039	3607928	43.4613	1.3514

## Discussion

### Comparison with other studies

In terms of the heterogeneity of landscape patterns, most of the existing studies focus on 2-D landscape analysis [32–38]. In this study, a metrics system of 3-D architectural landscape patterns was constructed to analyze the spatial heterogeneity of 3-D architectural landscape patterns in the main urban area of Xi’an. Researchers in other cities in China have also carried out studies on 3-D landscape patterns. For example, researchers in Shenyang, Qingdao, Dalian and other cities have used several architectural metrics to describe urban 3-D landscape patterns. Researchers usually select several metrics according to the needs to analyze the architectural landscape but seldom analyze the validity of the index selection [39–43]. For the selection of 3-D architectural metrics, commonly used indices such as AH, AV, BCR, CONTAG, and SHDI have been applied in architectural landscape research, which can reflect the changing characteristics of the architectural landscape [44]. In addition, some scholars created the HBR to help build a 3-D metrics system and found that the index has a certain effect on air quality [45]. This study introduces two new indices, the LHVC and BRI, to provide a more comprehensive understanding of the differentiation characteristics of the 3-D architectural pattern of the main urban area of Xi’an.

The United States, Italy, Japan, China and other countries have explored the scale effect of landscape patterns more [46–49]. Some researchers have analyzed the grain size effect of land use pattern changes at the type and landscape levels by changing the spatial grain size through the ratio dominance method of pixels [24]. In addition, some researchers used the grain size (resolution) of GIS data to evaluate the impact of grain size changes on landscape pattern metrics and cost-surface model output [25]. However, these studies mainly focus on the grain size effect of landscape patterns, and there were few studies on the extent effect, especially in Xi’an, where there is currently no such study.

This study analyzed the spatial heterogeneity of the landscape pattern for each functional land based on the analysis of the 3-D architectural landscape metrics differentiation. In similar studies, some researchers analyzed the spatiotemporal characteristics of the architectural landscape in the area using the 3-D information of buildings and found that the city was expanding vertically and that the intensity of urban land use had increased significantly year by year [23, 41]. Some researchers have used building-related landscape metrics to describe 3-D urban landscape patterns and used these metrics to describe urban characteristics, ecological conditions, and social significance [19]. The study found that these landscape indices can effectively reflect the relationship between the number, area, height, 3-D shape, and diversity of urban buildings. However, in Xi’an, there is no relevant research on the characteristics of 3-D landscape patterns.

In summary, compared with other studies, this study conducted a principal component analysis of landscape metrics and constructed a rigorous 3-D landscape pattern metrics system. Two new indices were introduced, LHVC and BRI, which enable a more comprehensive analysis of the spatial structure and heterogeneity of 3-D architectural landscapes and provide new possibilities for the selection of landscape indices in future studies. In addition, this study established a mathematical model of weighted changes in landscape metrics based on the CRITIC method to analyze the extent of changes in architectural landscape patterns and revealed the characteristic scales of 3-D landscape patterns in the study area. This study fills the gap in the research on the 3-D architectural landscape in Xi’an. Finally, this study comprehensively analyzed the spatial distribution characteristics of the landscape pattern in the study area at the micro and macro levels.

### Scale effect of the 3-D architectural landscape in the main urban area of Xi’an

The urban grid phenomenon of Xi’an is obvious, and the built-up areas have a chessboard design with have unique landscape ecological patterns. The boundaries of the first, second and third rings can reflect the expansion and evolution of the urban spatial structure of Xi’an and the characteristics of the urban landscape structure in the process of urban development. Spatial heterogeneity can reflect the complexity of the distribution pattern of landscape structure and functional space. It exists widely at multiple scales and is scale-dependent. For example, the measurement of landscape patterns are closely related to the selected scale of analysis. As shown in Fig 6, the values of SHDI, AH, BCR, HBR, and AV increase rapidly with increasing extent, and the scale dependence was strong. Between 6~8 km, the changes in these five landscape indices are not significant. Outside the range of 8 km, the values of these five indices slowly decrease and stabilize with increasing extent, and the scale dependence was weak. Within the range of 9 km, as the extent increases, the value of CONTAG decreases rapidly, and the scale dependence was strong. Outside the 9 km range, the landscape index gradually increases and tends to be stable with increasing extent, and the scale dependence was weak. In the range of 6 km, the value of LHVC changes drastically and irregularly, and the scale dependence was strong. Outside the range of 6 km, the index increases rapidly with increasing extent, and the scale dependence was strong. The value of the BRI increased continuously with increasing extent and had a strong scale dependence. From the above results, it is clear that the two newly introduced indices, LHVC and BRI, have a strong scale dependence compared to the other indices. For example, the values of these two indices change greatly with changes in extent.

The load plot in the PCA results can evaluate the "size or quality" of the variable’s influence on the sample. Therefore, Fig 5 shows that the load value of the LHVC is low, i.e., the information presented about the landscape is relatively low. BRI presents a larger amount of landscape information. Therefore, BRI was more effective than LHVC. Since the two new indices were sensitive to scales, they do not apply to studies of landscape patterns that require transformation between different scales. However, the new indices can be popularized and involved in the study of landscape patterns in a region to interpret landscape patterns from a wider range of perspectives.

Fig 7 shows that the weighted change value of the architectural landscape pattern index calculated based on the CRITIC method was consistent with the overall change trend of the entropy method, but the CRITIC method was more sensitive to changes in the architectural landscape pattern. Therefore, the weighted change model of the architectural landscape pattern index based on the CRITIC method was more robust. When we observe the weighted change plot of the landscape index based on the CRITIC method, we can see that the architectural landscape index changes more drastically in the range of 6 km and gradually decreases in the range of 6~8 km. Outside the 8 km range, the weighted change in the landscape index tends to be stable. This was because the range of 6 km was close to the location of the first ring road in Xi’an. Due to the unreasonable landscape composition in the first ring area and the mixed layout of residential and commercial areas, the architectural pattern has shown obvious changes. Between 6 km and 8 km was a transitional area from a mixed commercial and residential area to a predominantly residential area. Beyond 8 km, outside the second ring road, the land use structure tended to be stable. Therefore, the characteristic scale of architectural landscape patterns was approximately 8 km.

In addition, as shown in Fig 8, the architectural landscape is spatially positively correlated to all extents, with a high degree of spatial autocorrelation and small spatial differences. This indicates that buildings of the same type of height show an aggregated distribution in space. However, within 6 km, the spatial difference was relatively large, which was caused by a large number of old buildings in the first ring, the unreasonable layout between them and the new houses, and the fragmented arrangement of multiple height types of buildings. Beyond 8 km, the spatial difference of the architectural landscape tended to stabilize.

### Spatial differentiation characteristics of the 3-D architectural landscape in main urban area of Xi ’an

With the acceleration of urbanization, the population of Xi’an is increasing, especially in urban areas. The increase in population density has brought severe challenges to the resources in this area. To save land, cities have a tendency of employing vertical development, which has been shown by research in many cities [50–52].

As depicted in Fig 9, the high-value areas of the LHVC, BCR, SHDI, and BRI are Beilin District, Xincheng District, Lianhu District, and parts of Yanta District. As a result, it was concluded that the building density is higher, the roughness of the underlying surface is high, and the building height types are abundant and vary significantly in height in the Beilin, Lianhu and Xincheng districts. This is mainly because Beilin District, Lianhu District, and Xincheng District are the earliest built-up areas in Xi’an. The population density is high, there are many old buildings, commercial buildings are dense, and urban renewal is difficult, which leads to disorder and chaos in the building layout. The high values of AH are mainly distributed in the Baqiao and Yanta districts, and the low values are mainly distributed in Weiyang District. The HBR value of Baqiao District is higher, followed by Weiyang District, and there are also high-value areas of AV in these two districts. This was mainly because there are more newly developed buildings in Baqiao and Yanta, and the average height of the buildings is relatively high. In addition, Baqiao District has excellent ecological conditions and low population density, with only localized areas with large building volumes and high heights. The population density in Weiyang District is low, the average building height is relatively low, and a small number of large-volume buildings exist locally. The high-value areas of CONTAG mainly appear in Yanta, Weiyang, and Baqiao districts and are distributed near the third ring road of the city. From this, we know that the patch types of the architectural landscape cluster around the third ring road. This is due to the high land prices within the city’s first and second ring roads, and developers have focused construction in areas around the third ring road in Xi’an, with high-rise buildings dominating.

As seen from Table 4, the values of AH, BCR, and BRI of residential land are higher, and the value of CONTAG is lower. It is concluded that the residential land in the main urban area of Xi’an has a high average building height, high building density, large roughness of the underlying surface, and dispersed layout. This is mainly due to the high population density of the residential land in the main urban area of Xi’an and a large number of buildings and mainly high-rise buildings. The layout is often more flexible due to the need to meet the living needs of people in different regions. Commercial land has higher values of AH, BCR, AV and SHDI. It can be concluded that commercial land has a high building height, high building density, large volume, and rich building types. This is mainly because commercial land has a large flow of people compared to other lands and requires higher construction space utilization. The building types are full of changes. The values of LHVC and CONTAG of industrial land are higher, and the values of AH, AV, BCR, HBR, and SHDI are the lowest compared to other lands. Industrial land has a low building height and small size, low building density, fewer high-rise buildings, and fewer types of building patches. This is because industrial land is mainly located in suburban areas, with low population density, and land is not as scarce as in urban centers. Second, industrial land has various building types, such as factory buildings, living buildings, and office buildings, and the height varies greatly. In addition, there are many industrial parks in Xi’an, and industrial buildings tend to cluster. The values of HBR and AV are higher for transportation land, while the values of LHVC and BRI are lower. This is mainly because the transportation land has high pedestrian flow and the number of buildings is small and most of them are high-rise, resulting in a large proportion and volume of high-rise buildings. In addition, transportation buildings are generally public buildings with small height differences and low roughness of the underlying surface. The lower value of BCR for public service land is due to the public service land that usually has more public service facilities and a relatively lower building density.

## Conclusions

In this study, the 3-D landscape pattern analysis was applied to the landscape pattern study of the main urban area of Xi’an for the first time, and the spatial heterogeneity of the landscape pattern of buildings was analyzed. In this study, principal component analysis was used to conduct dimensionality reduction analysis on 12 3-D architectural landscape indices, and finally, 8 indices were selected, among which two indices, LHVC and BRI, were created for the first time, thus constructing the metrics system for 3-D architectural landscape patterns in the main urban area of Xi’an. In this study, a mathematical model of the weighted change in landscape metrics based on the CRITIC method was constructed to reflect the scale effect of a 3-D architectural landscape. The fractal dimension PAFRAC varies with the amplitude, which verifies the validity of the model. The model successfully reflects the characteristic scale of the 3-D landscape pattern and reveals that the characteristic scale of the 3-D landscape pattern in the main urban area of Xi’an is near 8 km. Through spatial correlation analysis, it was determined that the 3-D architectural landscape in the main urban area of Xi’an is spatially positively correlated, with a high degree of spatial autocorrelation and small spatial differences.

This study found that the characteristics of the 3-D urban landscape pattern are related to the natural geographic conditions, economic and production activities, and urbanization of Xi’an. The main urban area of Xi’an was densely built, creating a complex urban landscape. As different regions accommodate different urban economic, social and cultural functions, buildings vary in size, height, volume, density, morphological heterogeneity and vertical roughness. The architectural landscape pattern of Xi’an’s main urban area was disordered and chaotic within the second ring, and the phenomenon of patch type clustering near the third ring was closely related to geography, housing prices, traffic and other factors. Among the six administrative districts of Xi’an, Beilin District, Lianhu District and Xincheng District had higher building density, richer building height types and higher roughness of the underlying surface, which were the key areas for improvement in urban renewal. From the perspective of land use, residential land, commercial land, industrial land and public service land were the main urban functional land, among which residential areas had high building density and high height, industrial land had low building density and low height. The building density of public service land was low and the layout was centralized. The building density of commercial land was large and the volume was large. In the process of urbanization, how to make a reasonable layout of architectural space is an issue that government planning departments and urban designers need to pay attention to. From the perspective of urban landscape ecological design, we suggest that urban planning decision-makers in Xi’an should do a good job of integrating and transforming old and new buildings within the First Ring Road in the urban renewal process. It is necessary to focus on improving the layout of buildings and roads in Beilin District, Lianhu District, and Xincheng District to transform the scattered buildings to intensification to increase the utilization rate of land. In addition, the planning of commercial land should be strengthened so that the population density can be reasonably distributed, and finally, the various administrative regions should develop in a balanced manner. The reasonable layout of the architectural landscape has a profound impact on the quality of the urban ecological environment. This paper provides important support for further research on the mechanism of 3-D landscape pattern influence on the urban ecological environment.

## Supporting information

S1 Table Data of 3-D architectural landscape metrics for 2020. (XLS)

## References

[1] Scalenghe R, Marsan FA. The anthropogenic sealing of soils in urban areas. Landsc Urban Plan. 2009;90: 1–10. doi: 10.1016/j.landurbplan.2008.10.011

[2] Yu S, Yu B, Song W, Wu B, Zhou J, Huang Y, et al. View-based greenery: A three-dimensional assessment of city buildings’ green visibility using Floor Green View Index. Landsc Urban Plan. 2016;152: 13–26. doi: 10.1016/j.landurbplan.2016.04.004

[3] Zhang X, Wang Y, Li Z, Li W. Preliminary theory of three-dimensional urban landscape ecology. Acta Ecol Sin. 2007;27: 2972–2982. doi: 10.3321/j.issn:1000–0933.2007.07.039

[4] Wu J. Key concepts and research topics in landscape ecology revisited: 30 years after the Allerton Park workshop. Landsc Ecol. 2013;28: 1–11. doi: 10.1007/s10980-012-9836-y

[5] Du Preez C. A new arc–chord ratio (ACR) rugosity index for quantifying three-dimensional landscape structural complexity. Landsc Ecol. 2015;30: 181–192. doi: 10.1007/s10980-014-0118-8

[6] Wu J, Jelinski DE, Luck M, Tueller PT. Multiscale analysis of landscape heterogeneity: Scale variance and pattern metrics. Geogr Inf Sci. 2000;6: 6–19. doi: 10.1080/10824000009480529 11315667

[7] Wu J. Thirty years of landscape ecology (1987–2017): Retrospects and prospects. Landsc Ecol. 2017;32: 2225–2239.

[8] Botequilha Leitão A, Ahern J. Applying landscape ecological concepts and metrics in sustainable landscape planning. Landsc Urban Plan. 2002;59: 65–93. doi: 10.1016/S0169-2046(02)00005-1

[9] Yu S, Chen Z, Yu B, Wang L, Wu B, Wu J, et al. Exploring the relationship between 2D/3D landscape pattern and land surface temperature based on explainable eXtreme Gradient Boosting tree: A case study of Shanghai, China. Sci Total Environ. 2020;725: 138229. doi: 10.1016/j.scitotenv.2020.138229 32298895

[10] Wu Q, Guo F, Li H, Kang J. Measuring landscape pattern in three dimensional space. Landsc Urban Plan. 2017;167: 49–59. doi: 10.1016/j.landurbplan.2017.05.022

[11] Zhao X, Song Y, Hu Y, Liu Y. Optimizing Spatial Distribution of Residential Areas by Using Multi-Source Open Data. J Guangxi Norm Univ (Nat Sci Ed. 2020;38: 26–40. doi: 10.16088/j.issn.1001–6600.2020.01.004

[12] Listopad CMCS, Masters RE, Drake J, Weishampel J, Branquinho C. Structural diversity indices based on airborne LiDAR as ecological indicators for managing highly dynamic landscapes. Ecol Indic. 2015;57: 268–279. doi: 10.1016/j.ecolind.2015.04.017

[13] Baillard C, Maître H. 3-D reconstruction of urban scenes from aerial stereo imagery: A focusing strategy. Comput Vis Image Underst. 1999;76: 244–258. doi: 10.1006/cviu.1999.0793

[14] Mayer H. Automatic object extraction from aerial imagery—a survey focusing on buildings. Comput Vis Image Underst. 1999;74: 138–149. doi: 10.1006/cviu.1999.0750

[15] Jin X, Davis CH. Automated building extraction from high-resolution satellite imagery in Urban areas using structural, contextual, and spectral information. EURASIP J Appl Signal Processing. 2005;14: 2196–2206. doi: 10.1155/ASP.2005.2196

[16] Yu B, Liu H, Wu J, Hu Y, Zhang L. Automated derivation of urban building density information using airborne LiDAR data and object-based method. Landsc Urban Plan. 2010;98: 210–219. doi: 10.1016/j.landurbplan.2010.08.004

[17] Velastegui-Cáceres J, Rodríguez-Espinosa VM, Padilla-Almeida O. Urban cadastral situation in Ecuador: Analysis to determine the degree of proximity of the cadastral systems to the 3D cadastral model. Land. 2020;9: 1–20. doi: 10.3390/land9100357

[18] Chen Z, Xu B, Devereux B. Urban landscape pattern analysis based on 3D landscape models. Appl Geogr. 2014;55: 82–91. doi: 10.1016/j.apgeog.2014.09.006

[19] Liu M, Hu YM, Li CL. Landscape metrics for three-dimensional urban building pattern recognition. Appl Geogr. 2017;87: 66–72. doi: 10.1016/j.apgeog.2017.07.011

[20] Ahmed K N, Blackburn G A, Whyatt J D. Developing the desert: The pace and process of urban growth in Dubai. Comput Environ Urban Syst. 2014;45: 50–62. doi: 10.1016/j.compenvurbsys.2014.02.005

[21] Li Y, Zhu X, Sun X, Wang F. Landscape effects of environmental impact on bay-area wetlands under rapid urban expansion and development policy: A case study of Lianyungang, China. Landsc Urban Plan. 2010;94: 218–227. doi: 10.1016/j.landurbplan.2009.10.006

[22] Aguilera F, Valenzuela LM, Botequilha-Leitão A. Landscape metrics in the analysis of urban land use patterns: A case study in a Spanish metropolitan area. Landsc Urban Plan. 2011;99: 226–238. doi: 10.1016/j.landurbplan.2010.10.004

[23] Zhang P. Spatiotemporal features of the three-dimensional architectural landscape in Qingdao, China. PLoS One. 2015;10: 1–13. doi: 10.1371/journal.pone.0137853 26361016 PMC4567292

[24] Chen Y, Xie B, Li X. Spatial Grain Size Effect on Land Use Pattern Changes in Changsha City. Sci Geogr Sin. 2016;36: 564–570. doi: 10.13249/j.cnki.sgs.2016.04.010

[25] Corry RC, Lafortezza R. Sensitivity of landscape measurements to changing grain size for fine-scale design and management. Landsc Ecol Eng. 2007;3: 47–53. doi: 10.1007/s11355-006-0015-7

[26] Han L, Zhao J, Gao Y, Gu Z, Xin K, Zhang J. Spatial distribution characteristics of PM 2.5 and PM 10 in Xi’an City predicted by land use regression models. Sustain Cities Soc. 2020;61. doi: 10.1016/j.scs.2020.102329 32834929 PMC7293537

[27] Han L, Zhao J, Gu Z. Assessing air quality changes in heavily polluted cities during the COVID-19 pandemic: A case study in Xi’an, China. Sustain Cities Soc. 2021;70: 102934. doi: 10.1016/j.scs.2021.102934

[28] Mcgarigal K, Marks BJ. Fragstats: Spatial pattern analysis program for quantifying landscape structure. Dolores Po Box. USDA Forest Service—General Technical Report PNW 351; 1995. doi: 10.2737/PNW-GTR-351

[29] Zou ZH, Yun Y, Sun JN. Entropy method for determination of weight of evaluating indicators in fuzzy synthetic evaluation for water quality assessment. J Environ Sci. 2006;18: 1020–1023. doi: 10.1016/s1001-0742(06)60032-6 17278765

[30] Shi H, Li Y, Jiang Z, Zhang J. Comprehensive power quality evaluation method of microgrid with dynamic weighting based on CRITIC. Meas Control (United Kingdom). 2021. doi: 10.1177/00202940211016092

[31] Diakoulaki D, Mavrotas G, Papayannakis L. Determining objective weights in multiple criteria problems: The critic method. Comput Oper Res. 1995;22: 763–770. doi: 10.1016/0305-0548(94)00059-H

[32] Hou L, Wu F, Xie X. The spatial characteristics and relationships between landscape pattern and ecosystem service value along an urban-rural gradient in Xi’an city, China. Ecol Indic. 2020;108: 105720. doi: 10.1016/j.ecolind.2019.105720

[33] Kong F, Nakagoshi N, Yin H, Kikuchi A. Spatial gradient analysis of urban green spaces combined with landscape metrics in Jinan City of China. Chinese Geogr Sci. 2005;15: 254–261. doi: 10.1007/s11769-005-0038-2

[34] Luck M, Wu J. A gradient analysis of urban landscape pattern: A case study from the Phoenix metropolitan region, Arizona, USA. Landsc Ecol. 2002;17: 327–339. doi: 10.1023/A:1020512723753

[35] Shi J, Ma J. Analysis of Urban Green Space Landscape Pattern in Suzhou. Journal of Physics: Conference Series. 2020. p. 2034. doi: 10.1088/1742-6596/1549/2/022034

[36] Sun J, Xia H, Lan C, Xin K. A gradient analysis based on the buffer zones of urban landscape pattern of the constructed area in Guigang City, Guangxi, China. Acta Ecol Sin. 2006;26: 655–662. doi: 10.1016/S1872-2032(06)60012-7

[37] Yang R, Yang X, Xu D. Study on Change of Urban Green Space Landscape Pattern Driven by Construction of Eco-garden City. J Guangxi Norm Univ Sci Ed. 2020;38: 140–147. doi: 10.16088/j.issn.1001-6600.2020.06.016

[38] Zhang J, Shen H. Geo-spatial analysis and optimization strategy of park green space landscape pattern of Garden City–A case study of the central district of Mianyang City Sichuan Province. Eur J Remote Sens. 2020;53: 309–315. doi: 10.1080/22797254.2020.1725788

[39] Chen T, Liu M, Hu Y, Chang X. Differentiation characteristics of three-dimensional landscape pattern in Shenyang. Chinese J Ecol. 2015;34: 2621–2627. doi: 10.13292/j.1000-4890.2015.0238

[40] Fu F, Liu Z, Huang Q. Three-dimensional urban landscape pattern changes: A case study in the Central Business District of Futian, Shenzhen. Acta Ecol Sin. 2019;39: 4299–4308. doi: 10.5846/stxb201807021450

[41] Xu Y, Liu M, Hu Y, Li C, Xiong Z. Analysis of three-dimensional space expansion characteristics in old industrial area renewal using GIS and Barista: A case study of Tiexi District, Shenyang, China. Sustainability. 2019;11: 1860. doi: 10.3390/su11071860

[42] Yang J, Guo A, Xi J, Ge Q, Li X. Spatial-temporal differentiation of three-dimensional urban landscape pattern: A case study of Zhongshan District in Dalian. Acta Geogr Sin. 2017;72: 646–656. doi: 10.11821/dlxb201704007

[43] Zhang P, Hu Y, Xiong Z. Influence of location factor on the changes of three-dimensional architectural landscape in Tiexi District of Shenyang, Northeast China. Chinese J Ecol. 2012;31: 1832–1838. doi: 10.13292/j.1000-4890.2012.0300

[44] Zhang P, Hu Y. Variations of three-dimensional architecture landscape at different spatial scales. Chinese J Ecol. 2013;32: 1319–1325. doi: 10.13292/j.1000-4890.2013.0224

[45] Li D, Liu M, Li C, Hu Y. Relationship between urban atmospheric environment and surrounding two- dimensional and three-dimensional landscape pattern in China. Chinese J Appl Ecol. 2021;32: 1593–1602. doi: 10.13287/j.1001-9332.202105.015 34042353

[46] Bassa M, Chamorro L, Sans FX. Vegetation patchiness of field boundaries in the Mediterranean region: The effect of farming management and the surrounding landscape analysed at multiple spatial scales. Landsc Urban Plan. 2012;106: 35–43. doi: 10.1016/j.landurbplan.2012.02.005

[47] Liu Y, Liu X. Spatial scale effect of land use landscape pattern in Yongdeng County, Gansu Province, China. Chinese J Appl Ecol. 2016;27: 1221–1228. doi: 10.13287/j.1001-9332.201604.017 29732779

[48] Wang L, Yuan Y, Dong H, Huang X. Research on spatial scale effect of landscape pattern of land use in Wuhan City. World Reg Stud. 2020;29: 96–103. doi: 10.3969/j.issn.1004-9479.2020.01.2018370

[49] Zhao Y, Murayama Y. Effect of spatial scale on urban land-use pattern analysis in different classification systems: An empirical study in the CBD of Tokyo. Theory Appl GIS. 2006;14: 29–42. doi: 10.5638/thagis.14.29

[50] Koziatek O, Dragićević S. iCity 3D: A geosimualtion method and tool for three-dimensional modeling of vertical urban development. Landsc Urban Plan. 2017;167: 356–367. doi: 10.1016/j.landurbplan.2017.06.021

[51] Qin J, Fang C, Wang Y, Li G, Wang S. Evaluation of three-dimensional urban expansion: A case study of Yangzhou City, Jiangsu Province, China. Chinese Geogr Sci. 2015;25: 224–236. doi: 10.1007/s11769-014-0728-8

[52] Shi L, Shao G, Cui S, Li X, Lin T, Yin K, et al. Urban three-dimensional expansion and its driving forces—A case study of Shanghai, China. Chinese Geogr Sci. 2009;19: 291–298. doi: 10.1007/s11769-009-0291-x

